# Of Sound Mind and Body: Exploring the Diet-Strength Interaction in Healthy Aging

**DOI:** 10.3389/fnut.2020.00145

**Published:** 2020-08-28

**Authors:** Andrew T. Askow, Colleen F. McKenna, Allyson G. Box, Naiman A. Khan, Steven J. Petruzzello, Michael De Lisio, Stuart M. Phillips, Nicholas A. Burd

**Affiliations:** ^1^Department of Kinesiology and Community Health, University of Illinois at Urbana-Champaign, Urbana, IL, United States; ^2^Division of Nutritional Sciences, University of Illinois at Urbana-Champaign, Urbana, IL, United States; ^3^Neuroscience Program, University of Illinois at Urbana-Champaign, Urbana, IL, United States; ^4^Department of Cellular and Molecular Medicine, School of Human Kinetics, University of Ottawa, Ottawa, ON, Canada; ^5^Department of Kinesiology, McMaster University, Hamilton, ON, Canada

**Keywords:** dietary protein, resistance exercise, cognition, psychological well-being, power, physically strong

## Abstract

Strength is a vital component of healthy aging. However, “strength” comes in different forms (includes both physical and mental aspects) and can look different at various phases of adult life. Healthy eating and regular exercise are clearly important pillars for strength. This paper proposes a framework that underlines the value of protein foods and resistance exercise for aging strong.

## Introduction

Age-related decrements in muscle mass, strength, and power are associated with progressive declines in physical function (1–3). As such, strength and power are often characterized purely as physical qualities in the context of aging. However, there are other forms of strength that contribute to healthy aging. For example, mental strength, which we consider to be composed of both affective states (i.e., how one feels) and cognitive components, also plays an important role in the ability to adapt to the changes in demands of daily life throughout adulthood. As such, the term “strength” can be more broadly conceptualized as the ability of an individual to withstand and adapt to both physical and mental demand. Positive lifestyle behaviors, such as adhering to a healthy eating pattern and regular exercise, which have clear implications for promoting physical strength, could also exert a positive influence on mental strength (4). However, research examining the nutrition-exercise interaction for aspects of mental strength (e.g., affective-states and cognitive function) is limited. Furthermore, while we speculate there is an interplay between being mentally and physically strong, the mechanisms which underpin this proposed strength interaction remain poorly established.

**Figure 1:**
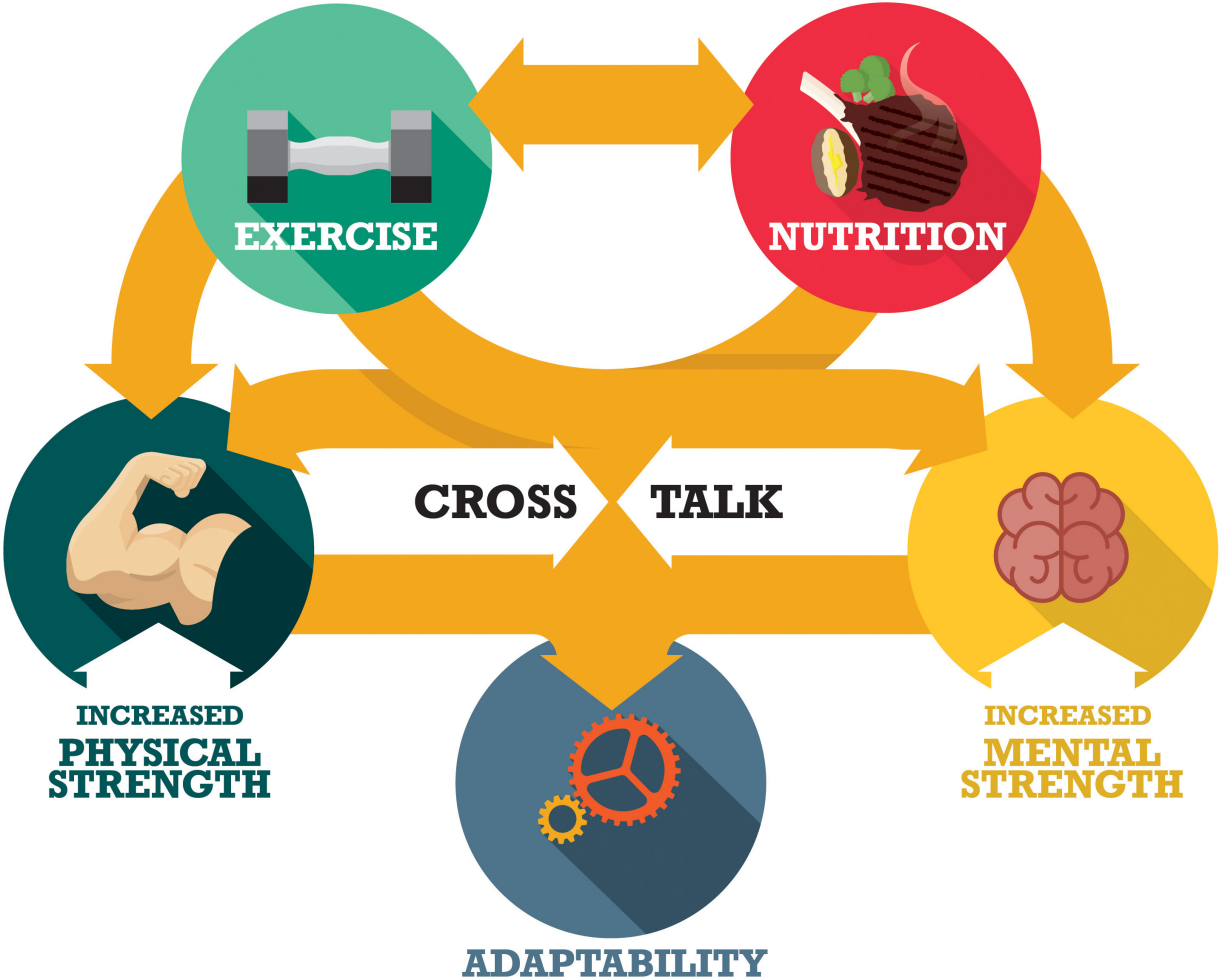
Physical and mental (cognitive, affective) strength have positive roles in supporting a healthy lifestyle and the ability to adapt to changing life demands throughout early, middle, and late adulthood. Positive lifestyle behaviors (e.g., healthy diet and regular exercise) exert a strong influence on ensuring the maintenance of strength in early adult life, and safeguard against declines in strength with advancing age. These lifestyle behaviors likely reinforce each other, but few studies have examined the nutrition-exercise interconnect especially in terms of mental strength. There is a proposed interplay between mental and physical strength. However, the mechanisms that underpin the strength interactions remain poorly defined.

Defining these mental and physical strength interconnections is important to maximize all aspects of strength early in adulthood and safeguard against declines with advancing age. From a healthy eating pattern perspective, it is evident that protein has central role within a healthy diet as evidenced by the fact that protein is the only macronutrient specifically represented on educational food guides (e.g., USDA's MyPlate) and lean body mass is negatively impacted when protein density of the diet is reduced in older adults (5). Moreover, it has been suggested that the assumed adequate protein intake for adults (0.8 g protein·kg^−1^·d^−1^), as defined by the United States' Recommended Dietary Allowance (RDA), may not be sufficient to offset age-related losses of physical strength (6, 7). Protein supplementation (i.e., eating above the protein RDA) has also been shown to be an effective strategy to potentiate the resistance training-induced skeletal muscle adaptive response (8, 9). Given the evidence that points to dietary protein intake and resistance training as modifiable lifestyle factors to support physical strength, it is important to consider how dietary protein may influence both physical and mental strength. Therefore, the aim of this paper is to discuss a theoretical framework by which to view exercise and nutrition as modifiable lifestyle factors for aging physically and mentally strong (Figure 1).

## Aging Strong: Physical Strength to Support Life-Long Health

The biological aging process is associated with decrements in skeletal muscle quantity and quality (i.e., the ability of a muscle to generate force proportional to its cross-sectional area) that negatively impact physical strength and muscular power (10). The incorporation of targeted exercise strategies, especially resistance training, has been shown to attenuate muscle mass and strength loss that occurs with aging (11, 12). Accordingly, past efforts have attempted to identify the optimal resistance training prescription to augment physical performance for all ages. Since many functional tasks are more dependent on power (i.e., force × velocity) than absolute strength [i.e., maximal force; (13)], it has been suggested that resistance training programs should primarily incorporate lighter loading schemes (≤60% of one-repetition maximum [1RM]) lifted at maximal velocity as these loads typically result in the greatest power output for a given exercise (14). However, this reductionist view of resistance training adaptations may discount the value of incorporating strength-focused training to enhance power and physical performance, especially for adults that are not already physically strong (15).

**Figure 2:**
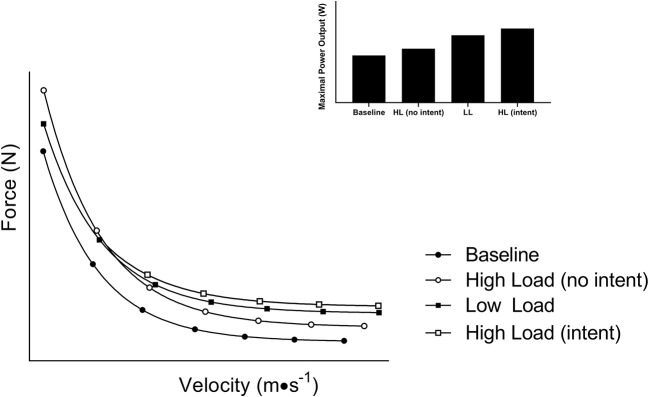
A schematic diagram of the shift in the force-velocity relationship after training with different loading paradigms. Training with heavier loads (higher force) typically shifts the curve upward while training with lighter loads (higher velocity) typically shifts the curve to the right. Both adaptations lead to an increase in power output. However, training with high loads at maximal volitional velocity yields a summative effect of high force and velocity leading to superior power gains.

At first glance, training at lighter loads (i.e., higher velocity) seems to result in more favorable power adaptation when compared to heavier loading, while the latter is favored for increasing maximal strength. To that end, meta-analyses comparing power training (e.g., lighter loading and high movement velocity) to conventional resistance training (e.g., heavier loading and slower movement velocity) suggest that power training is more effective to augment physical function and power output (16). Further, a number of reviews support the same conclusion with power training being the suggested approach to increase power and counteract age-related declines in physical function (17–19). However, much of this evidence is based on studies that factitiously limit concentric velocity of the heavier loading protocols by imposing a required cadence while instructing the power training group to move explosively. When high-load resistance training is completed with the intent to move explosively (e.g., rapid concentric), these differences disappear (20, 21). This likely can be explained by early work in this area which suggests that phenotypical shifts in muscle contractile characteristics after a training intervention are the result of intended, rather than actual, movement velocity (22). Given gains in strength (force) contribute to gains in power (23), training with higher loads, so long as repetitions are completed at maximum volitional velocity (or with intent to achieve high velocity), may have the potential to augment power output to a greater extent than low loading (Figure 2; open squares). Hence, building and maintaining an inherently strong muscle should not be overlooked given its pertinence for power performance and long-term independence to support healthy aging.

While resistance training is fundamentally the most potent stimulus to augment physical strength, there is a clear diet-strength interaction with regards to consuming enough daily protein to support more favorable muscle adaptations (8, 9). While early increases in strength are seemingly the result of neural adaptations and increased movement competence (24), increases in myofiber size contribute to long-term resistance exercise-mediated gains in strength (25). This muscle fiber growth is the result of successive periods of positive net muscle protein balance (NPB = muscle protein synthesis – muscle protein breakdown rates) that ultimately results in a significant deposition of contractile proteins, which is usually detectable within 6–7 weeks of the training program (26). While resistance exercise alone is capable of stimulating an increase in muscle protein synthesis rates, thereby improving NPB, the provision of exogenous amino acids during recovery from resistance exercise is required to shift NPB from negative into the positive. This dietary protein and resistance exercise interaction on NPB, albeit it is also impacted by intrinsic factors unique to the individual (27), provides that basis underpinning skeletal muscle adaptations. Despite the positive effects of dietary protein on the skeletal muscle adaptive response, there is a current disparity between assumed adequate intake (0.8 g protein·kg^−1^·d^−1^; RDA) and the observed “optimal” protein intake to support muscle health and physical strength (≥1.2 g·kg^−1^·d^−1^) (9). Further, the role of meal frequency or protein source (animal vs. plant) on modulating absolute daily protein need, and the subsequent effects on the skeletal muscle adaptive response, is not completely clear. For example, the consumption of large amounts of protein in a single meal (~40 g protein) does not lead to further increases in postprandial muscle protein synthesis rates, but instead results in excessive amino acid catabolism, when compared to the ingestion of ~20 g protein (28). Similarly, eating a spread distribution pattern of protein intake, which consists of smaller and more frequent meals throughout the day, has been shown to lead to greater muscle protein synthesis rates, and presumably daily net muscle protein accretion, when compared to eating the majority of daily protein (and energy) at dinner (29). Collectively, these results suggest that meal frequency should be a consideration when providing recommendations to optimize lean body mass and physical strength with advancing age. From a protein quality standpoint, it is possible to elicit a large rise in postprandial muscle protein synthesis rates, in terms of peak amplitude, when ingesting a plant based protein source (30). However, the consumption of larger amounts of plant-based protein derived from as single source (e.g., wheat) may be required to elicit a robust postprandial muscle protein synthetic response when compared to EAA-rich animal-based protein sources. Of course, in most vegetarian meal settings, complimentary protein pairings are generally used to improve plant-based protein quality and presumably the anabolic potential of a plant-based meal (31). Nonetheless, more work is required to better define the factors, such as protein quality and meal frequency, that influence “optimal” protein intake beyond absolute protein intake. What is noteworthy, however, animal protein foods are more effective than plant sources in meeting daily requirements of essential amino acids without the ingestion of excessive amounts of non-protein calories, and thus protein source/quality is an important consideration of optimal protein intakes. While it is relevant to acknowledge that eating animal sources of protein is not essential to meet daily protein intake recommendations, animal sourced protein foods contribute substantially to daily intake of a number of nutrients [e.g., vitamin D, vitamin B-12, calcium, etc.; (32)]. Hence, their inclusion in the diet can be used as an effective strategy to fulfill daily intakes of other dietary nutrients that are often lacking in the diets of older persons (33).

Certainly, it is important to highlight that not all studies support the notion that “high” animal based protein intakes have a positive benefit on resistance training-induced physical strength and muscle mass gain with age [c.f. (34, 35)]. For example, Holwerda et al. (35) demonstrated that the consumption of moderate (~1.2 g·kg^−1^·d^−1^) or higher protein intakes (~1.4 g·kg^−1^·d^−1^) both resulted in similar gains in skeletal muscle mass and physical strength after 12 weeks of progressive resistance exercise training in healthy older men. Indeed, both protein nutrition groups were consuming above the protein RDA and were regularly supplemented with high-quality animal based proteins (35). As such, it is not possible to firmly distinguish the impact of eating lower daily amounts of protein (or vegan diets) on resistance exercise-induced skeletal muscle adaptations in this particular study (35) and others (34, 36, 37).

Overall, the consumption of a variety of high-quality, nutrient dense protein foods (e.g., animal or using complimentary plant-based protein pairings) is necessary, while cognizant to energy intake requirements, to ensure the adequate intake of a number of nutrients as discussed elsewhere (32). This dietary strategy combined with strength-focused exercise training can likely be used as complimentary strategies to enhance physical strength and ensure adequate intakes of nutrients of concern (e.g., calcium, vitamin D, folate, or iron) with advancing age.

## Aging Strong: Mental Strength to Support Life-Long Health

The maintenance of mental strength (i.e., cognitive function and psychological well-being) is essential for independent living and active involvement in community life with advancing age (38). While both attributes of mental strength change with age, the dynamics of their change differ. Specifically, psychological well-being generally follows a *U*-shaped trend whereby subjective judgements of one's life decreases during middle adulthood before rebounding around 50 years of age and increasing thereafter (39). Conversely, cognitive function peaks during young adulthood and then steadily declines thereafter (40, 41). Nonetheless, evidence has established a link between impairments in cognitive function and psychological well-being and negative health outcomes (i.e., risk of chronic disease, mortality, and dependence). Thus, identifying strategies to support mental strength across the lifespan is important for long-term, healthy aging.

Similar to physical strength, physical activity and exercise training have been implicated as modifiable lifestyle habits which impart beneficial effects on cognitive function (42), and well-being (42, 43). Exercise may reduce the risk of developing neurodegenerative diseases (e.g., dementia) through increased neuroplasticity or improved cerebrovascular function (44, 45), and reduce the occurrence of mood disorders (i.e., anxiety or depression) by improving psychological well-being (43). While a disproportionate number of studies have investigated the effects of endurance training on mental strength, data from cross-sectional and intervention studies have suggested a positive link between strength (or strength training) and both cognition (46) and well-being (47). Moreover, evidence from meta-analyses suggests that participation in strength training results in improved cognitive performance (48) and addition of combined strength and endurance training potentiates cognitive improvement when compared to participating in endurance training alone (49).

Interestingly, the same dietary habits which enhance the effect of exercise for physical strength may also play a role in supporting mental strength. Higher dietary protein intake has been associated with better memory/recall (50), and a decreased risk of developing mild cognitive impairment or dementia, even when adjusted for other modifiable and non-modifiable risk factors (51). Further, dietary supplementation with essential amino acids (52) or a protein-dense nutritional drink (53) has been shown to significantly improve psychological well-being. However, results from randomized controlled trials on cognition are mixed. In one study, provision of daily milk protein, in combination with resistance training, for 24 weeks was shown to improve information processing speed among frail elderly adults while the non-supplemented group improved in attention and working memory (54). More recently, 24 weeks of habitual resistance and endurance exercise, based on a 10-point rating of perceived exertion intensity prescription, and lean red meat supplementation yielded similar increases in global cognition and executive function when compared to a control group (55). It should be noted, however, that this study was conducted against a dietary backdrop where all groups were consuming >1.2 g·kg^−1^·d^−1^ (55). Thus, the impact of a low(er) protein diet (e.g., 0.8 g·kg^−1^·d^−1^) on cognitive outcomes cannot be deciphered. Indirect evidence also suggests that nutrition and exercise strategies may support mental strength. Proteins are essential to maintain cellular integrity/function and specific amino acids (i.e., tryptophan and tyrosine) directly influence the synthesis of neurotransmitters (56). In addition, low protein intake is also associated with physical frailty (57), a known correlate of cognitive decline (58) and negative psychological well-being (59). Moreover, since skeletal muscle is an important regulator of whole-body metabolism, maintenance of muscle mass is important to protect against the development of chronic disease states which negatively affect mental strength. While the lack of randomized controlled trials investigating these relationships precludes definite conclusion, the theoretical framework proposed herein implicates an important role for protein foods and strength-focused exercise as positive lifestyle factors to support mental strength with advancing age.

## Potential Mechanisms Underpinning the Framework of Aging Strong

Despite the epidemiological evidence linking changes in physical and mental strength with age, the mechanisms underlying these interactions remain poorly understood. Complicating the investigation of these mechanisms is that the magnitude of physical and/or mental strength adaptations may be differentially impacted based on the exercise protocol (e.g., frequency, modality, intensity, and duration of training). Further, most of the evidence available is in patients or rodent models of neurological disease with limited studies in otherwise healthy aging. The majority of mechanistic work in this area has focused on the effects of endurance exercise on brain and cognition. Given that endurance and resistance exercise induce divergent skeletal muscle adaptations *via* different molecular mechanisms, they may also be expected to provide unique benefits to brain tissue and mental strength (i.e., affective and cognitive components).

The recent discovery of the myriad of factors released from contracting skeletal muscle, termed myokines, that contribute to the systemic benefits of exercise have been an active area of investigation to link muscle contraction to brain and cognitive adaptations (60). In the context of resistance training, brain-derived neurotrophic factors (BDNF), insulin-like growth factor-1 (IGF-1), and vascular-endothelial growth factor (VEGF) have been implicated as potential exercise-induced mediators of muscle-brain cross-talk. In general, findings from the limited number of studies investigating the muscle-brain link have been mixed (61). Several factors could be responsible for these mixed findings including inconsistent resistance exercise protocols, systemic vs. local (i.e., in muscle vs. brain) measures of these paracrine factors, and timing of blood/tissue collection. Recently, cathepsin B has been implicated as another exercise-induced paracrine factor involved in muscle-brain cross-talk (62). However, the response of cathepsin B to resistance exercise has not been investigated. In addition to myokines, the release of metabolites from contracting skeletal muscle (i.e., lactate), competition for mobilized endogenous fuel stores (i.e., glucose/lipids) between contracting skeletal muscle and active neurons, release of amino acids from damaged skeletal muscle as precursors to neurotransmitter synthesis, systemic hormones (i.e., cortisol/catecholamines), and retrograde signaling from postsynaptic motorneurons may also link muscle contraction to brain and cognitive adaptation (63). As such, a need exists for identification of novel biomarkers which could explain the muscle-brain connection, as well as determination of optimal resistance exercise protocols that can support physical and mental strength with advancing age.

## Perspective

A central theme of this paper is that “strength” needs to be redefined or broadened to include both physical and mental components, especially in the context of healthy aging. We speculate that lifestyle modifications such as increased habitual physical activity, strength-focused exercise, and high-quality protein consumption (>RDA) are good starting points to augment this framework of aging strong (see Figure 1). There is likely a stimulatory ceiling whereby increasing daily protein intakes above a certain threshold does not confer an additional benefit for mental or physical strength with resistance exercise training at a more advanced age. Certainly, a randomized controlled trial examining a range of protein intakes from the RDA to moderate to high combined with progressive resistance exercise training and various functional outcomes (e.g., physical strength and aspects of mental strength) is required to confirm our speculative aging strong framework. Moreover, the contribution of protein quality (i.e., animal vs. plant-based) or meal frequency/protein distribution on regulating the definition of “optimal” protein intakes for aging strong cannot be firmly stated at this time. There is, however, likely interplay between these protein intake variables that modulate an “optimal” protein recommendation (64). The presumed mechanistic links between physical and mental strength are somewhat elusive at the moment in healthy adults but provide opportunities for researchers and clinicians to favorably impact the effects of aging on physical strength, cognitive functioning, and psychological well-being.

## Author Contributions

AA, MD, NK, and AB drafted sections of the mini-review. NB, SJP, CM, and SMP provided critical revisions for content. All authors approved the final submission of the document, agree to be accountable for all aspects of the work, and made substantial contributions to the conception and design of the paper.

## Conflict of Interest

SMP has received research funding, honoraria for speaking, and travel expenses from the Beef Checkoff, through the National Cattlemen's Beef Association. NB, SJP, and NK have received grant funding from The Beef Checkoff. The remaining authors declare that the research was conducted in the absence of any commercial or financial relationships that could be construed as a potential conflict of interest.
